# Carbon mineralization pathways for carbon capture, storage and utilization

**DOI:** 10.1038/s42004-021-00461-x

**Published:** 2021-02-26

**Authors:** Greeshma Gadikota

**Affiliations:** School of Civil and Environmental Engineering, Robert Frederick Smith School of Chemical and Biological Engineering, Cornell University, Ithaca, NY USA

**Keywords:** Geochemistry, Carbon capture and storage, Sustainability, Process chemistry

## Abstract

Carbon mineralization is a versatile and thermodynamically downhill process that can be harnessed for capturing, storing, and utilizing CO_2_ to synthesize products with enhanced properties. Here the author discusses the advances in and challenges of carbon mineralization, and concludes that tuning the chemical interactions involved will allow us to unlock its potential for advancing low carbon energy and resource conversion pathways.

Advancing novel chemical processes to reduce the carbon intensity of our energy and resource conversion processes is one of our major scientific challenges. Diverse interventionist technologies to capture current CO_2_ emissions, reuse and store CO_2_ continue to be developed. One of the common themes across these different technologies is the role of inorganic solid carbonate transformations using anthropogenic CO_2_ and the development of predictive controls over these pathways. CO_2_ conversion to solid inorganic carbonates, also known as carbon mineralization, is a thermodynamically downhill route that can be adapted for integration with CO_2_-emitting energy and resource generating processes^1^. Despite the simplicity of the stoichiometric reactions describing the formation of Ca- or Mg-carbonates, complex chemo-morphological interactions result in non-monotonic kinetics of carbonate nucleation and growth.

## In situ and ex situ carbon mineralization

The availability of Ca- and Mg-bearing resources is crucial for implementing carbon mineralization pathways. About 10,000–1000,000 Gt of total carbon can be stored in naturally occurring mineral deposits^2^. Mine tailings resulting from nickel extraction^3^ and diamond production^4^ and chrysotiles (fibrous hydrated magnesium silicates) in asbestos^5^ are additional sources of alkalinity for carbon mineralization. Alkaline industrial residues such as fly ash (e.g., Class C fly ash produced by burning lignite or coal has a higher lime content compared to anthracite and bituminous coals that result in Class F fly ash with lower lime content^6^), cement kiln dust, steel slag, and red mud can store about 200–300 Mt of CO_2_ annually^2^. Co-generated CO_2_ and alkaline industrial residues can be reacted to produce Ca- or Mg-carbonates at the location of interest. Prior to mastering these transformations, it is important to consider how these pathways can be integrated into energy and resource conversion processes. CO_2_ generated from power plants or industrial sources can be captured, compressed, and stored in reactive geologic formations where CO_2_ in the fluid form mineralizes to produce water-insoluble calcium or magnesium carbonates, in a process known as in situ carbon mineralization. Alternatively, ex situ carbon mineralization pathways can be developed where the captured CO_2_ is mineralized into carbonates in engineered processes. Ex situ carbon mineralization can serve as a thermodynamically downhill strategy to capture and remove CO_2_ from energy and resource conversion processes^7,8^ that utilize fossil fuels and contribute to more than 70% of anthropogenic greenhouse gas emissions in the United States^9^. One of the advantages of ex situ processes is that products that can be employed for various applications such as inorganic carbonate can be produced, and therefore can be harnessed as CO_2_ utilization pathways.

## Integration of carbon mineralization with energy and resource conversion pathways

Various energy conversion pathways involving carbon-based resources (e.g., coal, natural gas, biomass, waste plastics) such as combustion, gasification, and anaerobic digestion are accompanied by the evolution of CO_2_. The emitted CO_2_ can be directly removed using Ca- and Mg-bearing precursors to produce Ca- and Mg-bearing carbonates. In the context of CO_2_ capture alone, calcium looping has been proposed in which calcium oxide is used to capture CO_2_ from flue gas streams to produce calcium carbonate. Calcium carbonate is heated at 900–950 °C to produce pure CO_2_ and regenerate calcium oxide^10^. The pure CO_2_ generated is then utilized and stored. The cycling efficiency of these approaches is dependent on the chemical and mechanical integrity of the oxide materials through multiple operating cycles.

As an alternative to calcium looping as a capture approach alone, capture and removal strategies via ex situ carbon mineralization can be implemented by using calcium and magnesium silicate or alumino-silicate rich minerals and industrial residues. The use of acids to dissolve the alkaline minerals and residues was proposed, followed by the sequential use of bases and then CO_2_ to produce calcium or magnesium carbonates. As an alternative to concentrated inorganic acids such as nitric or hydrochloric acid, mineral acids such as acetic acid or citric acid produced via microbial pathways have been proposed^11^. In addition to generating protons needed for dissolution, acetate or citrate ions serve as magnesium or calcium chelating agents, thus enhancing the availability of these cations for carbonate formation. Silica chelating agents such as metal organic frameworks^12^ and catechol have also been proposed to enhance dissolution of silicate minerals. Advancements in regenerable additives to enhance silicate dissolution at ambient temperature are needed.

Another challenge associated with carbon mineralization lies in enhancing the concentration of carbonate and bicarbonate ions needed for producing solid carbonates. Biological enzymes such as carbonic anhydrase were used to enhance CO_2_ hydration behavior^13,14^. As an alternative economical option to carbonic anhydrase, the use of recyclable aqueous CO_2_ capture solvents such as sodium glycinate^15^, monoethanolamine (MEA)^16,17^, and 2-amino-2-methyl-1-propanol^18^ are used to capture CO_2_. These CO_2_-loaded solvents enhance the supply of bicarbonate and carbonate ions, which are essential for precipitating Ca- and Mg-carbonates. The aqueous solvents are regenerated as the solid carbonates are precipitated. Near complete conversion of CaO to CaCO_3_ is achieved at 50 °C in 3 h using 30 wt% MEA or 1-M sodium glycinate with solid compositions of 15 wt% in well-mixed environments^15,17^. The inherent chemical regeneration of the solvents with carbonate formation occurs at 50–75 °C, which is significantly below the thermal regeneration of solvents at 100–120 °C.

Alternatively, Ca- and Mg-bearing sorbents can be directly utilized during the energy conversion process. One example is the enhanced conversion of H_2_ using alkaline sorbents such as Ca(OH)_2_ and Mg(OH)_2_ integrated with the water–gas shift reaction (WGSR) as represented by this overall reaction: CaO + CO + H_2_O = CaCO_3_ + H_2_. The WGSR is a versatile pathway to produce H_2_ and CO_2_ from CO and water, the products of gasification or reforming of carbon-based resources including biomass^19^ as represented by this reaction: CO + H_2_O = CO_2_ + H_2_. Given this challenge, conventional modes of operation involve two catalytic systems, one operating between 310 and 450 °C and another between 200 and 250 °C to achieve high conversion^20^. The pressure is in the range of 20–30 bar. Integrating alkaline sorbents with the WGSR for concurrent CO_2_ capture shifts the thermodynamic equilibrium toward enhanced H_2_ production. Proposed advances include the direct use of calcium or magnesium silicates to produce H_2_ and calcium or magnesium carbonates. This approach eliminates the intermediate step to produce Ca- and Mg-hydroxides or oxides. The hypothesis that the two-step catalytic WGSR can be replaced by a single-step approach by integrating with carbon mineralization is being probed^21^. The promise in the direct utilization of Ca- and Mg-bearing silicates emerges from evidence showing complete conversions of calcium silicate (CaSiO_3_) and higher than 80% conversion of magnesium silicate (Mg_2_SiO_4_) to their respective carbonates at temperatures in the range of 150–200 °C^17,22–24^, which align with the WGSR conditions. The thermodynamic feasibility of this pathway is represented by the following reactions. Equation (1) below represents syn gas generation. Equations (2)–(4) represent the carbon mineralization of magnesium silicate. The overall combination reaction in Eq. (5) shows that coupling carbon mineralization with the WGSR is overall thermodynamically favorable.

1 $$2{\mathrm{CO}} + 2{\mathrm{H}}_2{\mathrm{O}} = 2{\mathrm{CO}}_2 + 2{\mathrm{H}}_2\,(\Delta {\mathrm{H}} = - 82.4\,{\mathrm{kJ}}/{\mathrm{mol}})$$

2 $${\mathrm{Mg}}_2{\mathrm{SiO}}_4 + 2{\mathrm{H}}_2{\mathrm{O}} = 2\,{\mathrm{Mg}}\left( {{\mathrm{OH}}} \right)_2 + {\mathrm{SiO}}_2\,(\Delta {\mathrm{H}} = - 99.7\,{\mathrm{kJ}}/{\mathrm{mol}})$$

3 $$2{\mathrm{Mg}}\left( {{\mathrm{OH}}} \right)_2 = 2{\mathrm{MgO}} + 2{\mathrm{H}}_2{\mathrm{O}}\,(\Delta {\mathrm{H}} = 162.4\,{\mathrm{kJ}}/{\mathrm{mol}})$$

4 $$2{\mathrm{MgO}} + 2{\mathrm{CO}}_2 = 2\,{\mathrm{MgCO}}_3\,(\Delta {\mathrm{H}} = - 235.6\,{\mathrm{kJ}}/{\mathrm{mol}})$$

5 $${\mathrm{Mg}}_2{\mathrm{SiO}}_4 + 2{\mathrm{CO}} + 2{\mathrm{H}}_2{\mathrm{O}} = 	\,2{\mathrm{MgCO}}_3 + {\mathrm{SiO}}_2 \\ + 	2{\mathrm{H}}_2\,(\Delta {\mathrm{H}} = - 255.3\,{\mathrm{kJ}}/{\mathrm{mol}})$$

Another complimentary strategy is to produce bio-hydrogen with carbon removal via mineralization. Renewability, the potential for distributed energy generation, and the opportunity to convert heterogeneous residues such as food waste to useful fuels and products are unique to biomass as an energy resource^25^. The integration of carbon mineralization with renewable biomass resources has been proposed to accelerate H_2_ production with the inherent conversion of organic carbon present to inorganic carbonates, as represented by the following reaction^19^:

6 $${\mathrm{C}}_{x}{\mathrm{H}}_{y}{{\mathrm{O}}_{z}} + {x}{\mathrm{CaO}} + \left( {2{x}} - {z} \right){\mathrm{H}}_2{\mathrm{O}} \to {x}{\mathrm{CaCO}}_3 + (2{x} - {z} + 0.5{y}){\mathrm{H}}_2$$

**Fig. 1:**
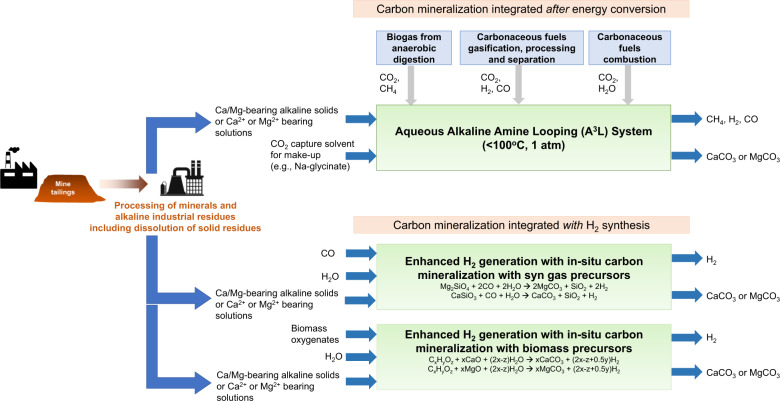
Schematic representation of the integration of carbon mineralization pathways and energy conversion processes. The aqueous alkaline amine looping reactor captures CO_2_ from flue gas using aqueous solvents such as amines and amino acid salts, and the high concentrations of carbonate and bicarbonate ions react to produce calcium or magnesium carbonates, with the inherent regeneration of the solvent. This reactor is integrated with various CO_2_ emitting energy conversion processes. Carbon mineralization can be integrated with the water–gas-shift reaction or biomass conversions to enhance H_2_ formation and remove CO_2_ as calcium or magnesium carbonates.

The standard heats of reaction for various biomass oxygenates for Eq. (6) are shown in Table 1. This approach has the potential to meet distributed energy needs using a clean energy carrier with inherent carbon removal. Alkaline sorbents such as CaO or Ca(OH)_2_ enhance the cleavage of C–C bonds and capture the generated CO_2_ concurrently as the reaction progresses. For example, more than 90% conversion of cellulose to H_2_ occurred above 500 °C when mixed with stoichiometric proportions of solid Ca(OH)_2_ and in the presence of Ni/ZrO_2_ catalyst^26^. As opposed to generating oxides and hydroxides of Ca and Mg from carbonates, alternative routes of synthesizing these materials from silicate precursors need to be investigated. Figure 1 is a schematic representation of the integration of carbon mineralization pathways with flue gas streams, biomass utilization, and syn gas generation.

**Table 1:** Standard heats of reaction (kJ/mol) for the conversion of biomass oxygenates to hydrogen with carbon removal through the following reaction: C_*x*_H_*y*_O_*z*_ + *x*CaO + (2*x* − *z*)H_2_O → *x*CaCO_3_ + (2*x* − *z* + 0.5*y*)H_2_^19^.

Biomass oxygenate	Standard heat of reaction (kJ/mol)
Methanol	−92
Ethanol	−186
Glycerol	−411
Butanol	−325

In the context of resource utilization, determining the fate of hazardous elements co-present with alkaline industrial residues is an important consideration. Carbon mineralization can reduce the mobility of elements of potential environmental concern, such as Zn, Cu, and Pb as amphoteric constituents and facilitate the partial immobilization of Cr for carbonated Ca-rich slag^27^. This approach enables the reuse of alkaline materials and reduces the cost of landfilling. Incorporating compositions as low as 10 wt% of carbonate-bearing steel slag in construction materials has shown to enhance the compressive strength from about 33–50 MPa^28^. CO_2_ curing of synthetic calcium silicate to produce high strength carbonate-bearing construction materials has now been translated into practice^29^.

Advancing the science of carbon mineralization also has a translational impact on informing the fate of CO_2_ injected into subsurface formation bearing reactive calcium and magnesium silicates, also known as in situ carbon mineralization. Field-scale studies such as the injection of pressurized CO_2_ in reactive basalt formations in Iceland^30^ and in Washington State^31^ demonstrated that the Ca- and Mg-silicate constituents of basalt converted to Ca- and Mg-carbonates over the course of a few years. These geochemical conversions were mimicked at representative subsurface conditions of elevated temperature (e.g., 100–200 °C^22–24^) and CO_2_ partial pressures (e.g., 50–200 atm^22–24^) with high surface area particles and sizes in the range of 5–100 μm. Near complete conversion of calcium silicate minerals such as wollastonite (CaSiO_3_) and magnesium silicate minerals such as olivine ((Mg,Fe)_2_SiO_4_) were achieved on reacting for 3–6 h^22–24^. In contrast, the reactivities of alumino-silicate bearing minerals and rocks do not exceed 50% at comparable reaction conditions^22^. Carbonates and silica are co-present in the materials obtained after reacting with CO_2_ at conditions relevant to the subsurface environments. Silicate weathering and carbonate formation mechanisms in the subsurface environments are needed to predict the fate of CO_2_ injected into reactive subsurface environments. The silica content and its reactivity influence the utilization potential of alkaline industrial residues and minerals.

## Outlook

Despite the advancements made in producing Ca- and Mg-bearing carbonates from anthropogenic CO_2_, several scientific challenges remain. The chemical compositions and morphologies of alkaline sources such as naturally occurring minerals or alkaline industrial residues are heterogeneous. The influence of silica and iron constituents on the mechanisms and rates of carbonate formation in diverse fluidic environments need to be elucidated. Often, the kinetics of carbonate formation starting from multicomponent precursors can be non-monotonic and developing predicting controls remains a challenge.

As an alternative to existing acid and base consumptive methods to produce Ca- and Mg-bearing carbonates, there is a need for solvents or sorbents that can supply CO_2_ and can be regenerated in situ as carbonates are formed. Novel chemical pathways to synthesize high purity, nano-scale, and meso-scale Ca- and Mg-carbonates from anthropogenic CO_2_^28,32^ with few additional unit operations and regenerable solvents are needed. These materials have wide-ranging applications in the paper industry and as filler materials.

Carbonates from anthropogenic CO_2_ are typically formed in multiphase environments. Characterizing the evolution of the fluid chemistry and the structure and morphologies of carbonates and development of the underlying mechanisms requires the use of novel in operando cross-scale and multimodal characterization methods such as X-ray and neutron scattering, spectroscopy, and tomography at representative temperature and pressure conditions. Advanced characterization of the carbonate products is needed based on the end use of these materials. For example, the use of metastable hydrated magnesium carbonate as algae feed requires the quantification of these phases in the end product^33^. When considering the use of carbonate-bearing construction materials, linking the carbonate phases and content to the mechanical strength of these materials is crucial.

A fundamental understanding of the dissolution and carbonate formation mechanisms in silicate minerals has a translational impact on our understanding of the storage of CO_2_ in subsurface geologic formation. Rapid conversion of the injected CO_2_ into water-insoluble carbonates reduces the mobile fraction of CO_2_ and the need to monitor the fate of mobile CO_2_. Dynamic changes in the dissolution and carbonate formation behavior along with the associated changes in the pore and fracture morphology need to be accounted for when evaluating the fate of CO_2_ in the subsurface environments. Thus, advancing the science of carbon mineralization in subsurface and engineered systems is integral to our carbon management efforts.
